# Readaptation Treatment of Mal de Debarquement Syndrome With a Virtual Reality App: A Pilot Study

**DOI:** 10.3389/fneur.2020.00814

**Published:** 2020-08-18

**Authors:** Sergei B. Yakushin, Reilly Zink, Brian C. Clark, Chang Liu

**Affiliations:** ^1^Department of Neurology, Icahn School of Medicine at Mount Sinai, New York, NY, United States; ^2^Ohio Musculoskeletal and Neurological Institute (OMNI), Ohio University, Athens, OH, United States; ^3^School of Electrical Engineering and Computer Science, Ohio University, Athens, OH, United States; ^4^Department of Biomedical Sciences, Ohio University, Athens, OH, United States

**Keywords:** Mal de Debarquement syndrome, velocity storage, readaptation, rocking, swaying, bobbing

## Abstract

Mal de Debarquement syndrome (MdDS) is composed of constant phantom sensations of motion, which are frequently accompanied by increased sensitivity to light, inability to walk on a patterned floor, the sensation of ear fullness, head pressure, anxiety, and depression. This disabling condition generally occurs in premenopausal women within 2 days after prolonged passive motion (e.g., travel on a cruise ship, plane, or in a car). It has been previously hypothesized that MdDS is the result of maladaptive changes in the polysynaptic vestibulo-ocular reflex (VOR) pathway called velocity storage. Past research indicates that full-field optokinetic stimulation is an optimal way to activate velocity storage. Unfortunately, such devices are typically bulky and not commonly available. We questioned whether virtual reality (VR) goggles with a restricted visual field could effectively simulate a laboratory environment for MdDS treatment. A stripes program for optokinetic stimulation was implemented using Google Daydream Viewer. Five female patients (42 ± 10 years; range 26–50), whose average MdDS symptom duration was 2 months, participated in this study. Four patients had symptoms triggered by prolonged passive motion, and in one, symptoms spontaneously occurred. Symptom severity was self-scored by patients on a scale of 0–10, where 0 is no symptoms at all and 10 is the strongest symptoms that the patient could imagine. Static posturography was obtained to determine objective changes in body motion. The treatment was considered effective if the patient's subjective score improved by at least 50%. All five patients reported immediate improvement. On 2-month follow-ups, symptoms returned only in one patient. These data provide proof of concept for the limited-visual-field goggles potentially having clinical utility as a substitute for full-field optokinetic stimulation in treating patients with MdDS in clinics or via telemedicine.

## Introduction

Mal de Debarquement syndrome (MdDS) is a debilitating phantom sensation of motion that generally occurs within 48 h after prolonged transportation (motion triggered, MT) or with no specific motion preceding it (spontaneously occurred, SO) (1). The most frequent motion sensations are of rocking (forward back), swaying (side-to-side), bobbing, walking on a trampoline, or gravitational pull in a specific direction. Motion sensations are frequently accompanied by psychosomatic symptoms such as brain fog, fullness of ears, heavy head and heavy leg sensations, fuzzy vision, fatigue, and high sensitivity to fluorescent lights, computer screens, and unsteadiness in crowds (2). The distinct difference between MdDS and the other vestibular disorders is that symptoms of MdDS are temporarily relieved by passive motion (1).

Head rotation is sensed by hair cells located at the ampulla of the semicircular canals of the inner ear. When the head is rotated at a constant velocity, primary vestibular afferents sense this rotation for ≈4 s from the rotation onset (3–5). At the same time, when the subject is rotated in darkness at a constant velocity, this rotation induces eye nystagmus that lasts 12–20 s (6), which is longer than the response time of the primary afferents. Thus, information about head velocity must be stored in the brain to produce such an extended eye response. In the 1970s, two groups of scientists simultaneously came to the concept that they called velocity storage mechanism (integrator) (7, 8). Despite minor differences in the two proposed models, both suggested that velocity information is temporarily stored in the brain. Because nystagmus dies away within 12–20 s, the integrator was modeled with a leak of stored velocity signal. Both models assumed that the vestibulo-ocular reflex (VOR) is composed of 3-neuronal (direct) VOR pathway that is more or less independent of the polysynaptic velocity storage (indirect) pathway.

Over the next three decades, the spatial properties of the velocity storage were intensively investigated (9–11). One of the most important findings was that velocity storage could be identically activated either by head rotation or by optokinetic stimulation (OKS) (7). If the subject is kept in darkness in the upright position after the horizontal optokinetic nystagmus (OKN) was induced, OKN is followed by horizontal optokinetic after nystagmus (OKAN), which dies away with the time constant of velocity storage (12, 13). A second important finding was that when horizontal OKN is induced with the subject tilted relative to gravity, it induces vertical or torsional OKAN components, depending on head orientation relative to gravity. As a result, when nystagmus is initiated off the vertical axis, the axis of eye rotation (eigenvector) during OKAN tends to align with the axis of gravity (12, 13).

OKN through velocity storage mechanism complements the function of the vestibular system by prolonging eye movement response to constant velocity of rotation in light (9, 10). While gain (slow-phase eye velocity/stimulus velocity) of the vestibular function is optimal at high frequencies (14–16), the gain of OKN is optimal at low frequencies and low peak velocities (17).

Lesion of the foveal area in primates did not affect the profile of slow-phase eye velocities of OKN (18). This implies that true OKN and velocity storage are generated by the visual periphery (19). As such, it is logical to assume that full-field OKN stimulation should be stronger than visual stimulation of the restricted visual field. Indeed, it has been shown that edges of the visual field affect OKN (20). Thus, OKS with restricted visual field may not be as effective as a full-field OKN. While the size of the visual field is a factor (21), however, it is less relevant for low-speed OKN, which is commonly used for MdDS treatment (22, 23). Human horizontal visual field is about ±105°, while vertical is only 50 and 70° when looking up and down, respectively (24). It was previously demonstrated that goggles with limited field (±44° horizontally and ±36° vertically) can adequately activate velocity storage (13). The visual field of our virtual reality (VR) goggles was approximately ±45° horizontally and vertically, which is comparable with what was previously used to activate velocity storage (13). Thus, the purpose of this project was to determine whether limited-field OKN produced by VR goggles could substitute for full-field OKN in successful treatment of MdDS. The velocity storage integrator is a network of neurons between left and right rostral medial and superior vestibular nuclei (25). Neurons that code velocity storage utilize GABA_B_ as a transmitter (26–28). Their firing rate is not related to eye movement *per se* but is related to head velocity. This is why they are called vestibular-only (VO) neurons. Their firing rate, however, is modulated by OKN (29–31). VO neurons do not project directly to the oculomotor plant (32) but rather are a part of the polysynaptic (indirect) VOR pathway (31). Many of them project to the spinal cord and are part on the vestibular postural control (33).

The gain (eye velocity/head velocity) of the direct VOR can be modified (adapted) within minutes by changing the visual feedback in response to head rotation (34–36). However, induced changes are reversed back within the same time if conditions change (37, 38). VOR gain adaptation can be induced with a specific context (39–41). Contextual adaptation is more prolonged, and gain changes induced in 1 h can last for several days (42, 43).

Similar to the direct 3-neuronal VOR pathway, horizontal (yaw), vertical (pitch), and torsional (roll) components of velocity storage can be adapted (44). This is a key foundational premise for the MdDS treatment approach proposed by Dai et al. (2, 45). The full-field OKN induces a sensation of body rotation in the direction opposite to stripes' motion (vection) that is indistinguishable from actual rotation in light with stripes stationary in space (46). It was recently demonstrated that amounts of vection and velocity storage are directly correlated (47). Thus, according to VOR readaptation hypotheses, when the ship is turning relative to the coastline, it induces horizontal OKN (yaw axis rotation), if the passenger is facing the ship's direction of motion. At the same time, all ships are oscillating side-to-side at ≈0.2 Hz, which is a dominant frequency of water oscillation in the ocean (48). The ship's side-to-side oscillations induce head oscillations about the naso-occipital (roll) axis. Simultaneous head rotation about 2-axis according to Euler's rotational theorem is inducing rotation about third (pitch) axes (49). Thus, if the passenger is facing the motions, oscillations about pitch axis can be stored in the velocity storage part of the VOR pathway (44, 50). Similarly, if the patient stands sideways to the ship's long axis, oscillations in roll can be stored as a contextual learning. This stored information is interpreted by the brain as a sensation of body rocking or swaying, when the passenger steps off the ship.

It was proposed that permanent changes in VOR that occurred in MdDS patients after traveling could be reversed by activating the polysynaptic velocity storage path of VOR with a full-field OKN, while the head is oscillated side-to-side or up–down. Head oscillations side-to-side induce cross-coupled sensations of head motion forward–back (44). Head motion up–down induces sensations of swaying side-to-side. Thus, to treat rocking, the velocity storage should be activated in the direction that is opposite to what was experienced during traveling. If the stored sensation is rocking, the head during the treatment should be oscillated side-to-side. If the sensation is side-to-side swaying, the head should be moved up–down (44). If the head is oscillated at the frequency of phantom motion, in both cases, this treatment induces sensations of motion that are out of phase with the phantom motion. As a result, the induced and stored sensations should cancel each other, and phantom sensation is reduced or cured.

## Methods

### Study Participants and Study Overview

We sought to conduct a proof-of-concept study examining the utility of this limited-field OKN VR system in delivering VOR readaptation treatment to patients with MdDS. To this end, five women volunteered to undergo VOR readaptation treatment using the limited-field OKN VR system. Over the last 5 years, the Vestibular Treatment Center of Mount Sinai has provided treatment of MdDS daily, with about five to six applications each week. Patients for the present study were selected from this applicant database. Local patients were preferred because they could be called back for the original treatment if the VR goggle treatment did not work. Thus, four out of five patients in this study were local. Only female patients were considered because MdDS is more common among females than among males. All study participants reported experiencing MdDS symptoms for at least 1 month, and four of the five subjects attributed their MdDS symptoms to passive motion exposure (e.g., a cruise). Additional characteristics of the study participants are described in the results. Three of the study participants underwent four treatment sessions, one underwent seven, and another one underwent two treatment sessions. The Mount Sinai Institutional Review Board (IRB) approved the protocol, and all subjects provided written informed consent.

On the first treatment day, spontaneous nystagmus, static post-urography, and Fukuda tests were administered to determine the direction of the OKN visual stimulus and the frequency of the head oscillation as previously described (2, 45). Treatment was delivered with OKN at 5°/s.

### Development and Implementation of the Limited-Field OKN VR System

We developed a limited-field OKN VR system using a mobile Google Daydream app using the Unity3D graphics engine, which we have previously described (51). This app is not currently publicly available, but researchers interested in utilizing this app can contact the Ohio Musculoskeletal and Neurological Institute at omni@ohio.edu for more information. In brief, stripes moving left, right, up, and down were implemented in four different programs. The speed of stripes could be adjusted in 5°/s increments from 0 to 20°/s. During the treatment, stripes' orientation to gravity remained unchanged despite head motions. Initially, Google Cardboard was selected as the development hardware for the intended MdDS treatment app. A prototype was implemented using Open Graphics Library for Embedded Systems (OpenGL ES) in the native Android Software Development Kit (SDK). While the prototype app was functional, we found out that many Google Cardboard goggles on the market were hard to secure firmly on one's head and had difficulty avoiding slight movement while the head was in motion. In the second phase of the project, to provide a better VR user experience, we switched to Google Daydream viewers and used Unity3D, a mobile graphic engine, to develop a Daydream VR mobile app. Key considerations in the design were (1) adjusting the environment to extend upward in an almost cone-like shape to accommodate the tendency for users to look upward when in a virtual space; (2) placing the lighting in the same position as the camera; (3) not having a “floor,” such that the lines result in a sphere-like setting; (3) having a simple and easy-to-use interface; (4) developing four different applications including clockwise vertical lines, counterclockwise vertical lines, upward horizontal lines, and downward horizontal lines; and (5) accompanying audio with each speed that is easily adjustable, so the user will always be aware of the current rotation speed in degrees per second.

Google Daydream only runs on mobile phone models that are explicitly identified as Daydream-ready phones to ensure that they possess sufficient processing power in Daydream mode to reduce latency and prevent nausea. Furthermore, the 3D model used in our program is simplistic, with only simple textures and low polygon counts, much simpler than a typical mobile 3D game scene. On our test devices—Google Pixel 2 and Pixel 2 XL—there were no perceptible latency issues at all.

During some treatment sessions, patients' heads were manually passively moved side-to-side to treat the sensation of rocking (forward–backward) (45) or up–down to treat swaying (side-to-side) (2). Manual head deviation from upright in each direction was ≈20°. To maintain repeatable motion, we played metronome music, which comprised seven notes on a musical scale (heptatonic scale) played in ascending and descending order of pitch (A–B–C–D–E-F#–G#–A). During treatment, the patients sat in a stationary chair with the head in the upright position with their eyes closed. The musical metronome was started, and the head was moved when the ascending scale reached tone D. At the time of tone A, the head would be in a maximally deviated position, and as the scale went in descending order to low A, the head would reach maximal deviation from upright in the alternate direction. The metronome was programmed to play over a specific time. When the music stopped, head motions stopped too, and the patient was asked to close their eyes, while the Oculus frame was removed from the subject's head.

To determine the direction of stripes for rocking and swaying, we performed a Fukuda step test (52, 53). If the Fukuda was positive (rotation >20°), stripes were moved in the direction opposite to body rotations. Some patients reported sideways gravitational pull sensations. For these patients, the stripes were implemented in the direction opposite to the pull.

### Static Post-urography and MdDS Severity

Static post-urography was obtained with eyes open and closed with feet 27 cm apart using a Wii board (Nintendo Inc.). Data were wirelessly transferred to the computer. Fast Fourier transform was used to determine the frequency of rocking and swaying (45). To compare postural stability after individual treatments, the displacement of center of pressure (COP) over a 20-s period was computed as well as the root mean square (RMS) of the postural displacement along *X*- (xRMS) and *Y*-axes (yRMS) (2).

Symptom severity was self-scored by the patients on a scale of 0–10, where 0 is no symptoms at all and 10 is the strongest symptoms that the patient could imagine (54). Symptom self-scoring was obtained before and after each treatment session. When treatment was completed, scores prior to and after the treatment were compared. We *a priori* considered that treatment would be considered successful if a patent's improvement in pretreatment and post-treatment self-scores was >50%.

### Treatment Procedures

#### Treatment Approach for Rocking and Swaying

During the treatment, patients sat in a regular (not revolving) chair. The goggle frame was adjusted to a comfortable level prior to treatment. The software was run by the researcher. The patient was then asked to close their eyes and place the goggle frame on their head. The metronome software was run on a computer. Then the patient was asked to open their eyes while the researcher provided passive head motions at the frequency of the metronome. We first moved the head at the frequency of body oscillation. If effective, the head was also moved at lower frequencies. Typically, frequencies of 0.2 Hz for 1 min, 0.1 Hz for 3 min, and 0.05 Hz for 5 min were considered. If the body was rocking (forward–back), the head was moved side-to-side. If the body was swaying (side-to-side), the head was rocked (up–down) (44).

#### Treatment Approach for Sideways Gravitational Pull

During this treatment, the patient sat as described above. The head, however, remained stationary upright. If the patient complained of a sideways gravity pull, the stripes were moved in a direction opposite to the pull direction. If the patient complained of sensations of pulling backward or walking on a soft ground or a sensation that their body is too light, then stripes going upward were used. If the patient complained of being pulled forward, walking on a moving sidewalk at the airport sensation, or body heaviness, the stripes were moved down.

### Statistical Analysis

For this proof-of-concept study, we sought to compare changes before and after treatment. Here, we used the Wilcoxon matched-pairs signed-rank test to compare two groups of data. The Pearson chi-square test was used for categorical data. A preset alpha level of significance of 0.05 was required for statistical significance. All statistical analyses were performed using SPSS.

## Results

### Descriptive Characteristics of the Study Participants

Five female patients aged 42 ± 10 years (range 26–50) were treated for MdDS with VR goggles (Table 1). Two patients had their symptoms triggered immediately after several days on cruises, one after several hours of boarding, and one immediately after a car ride. There was no specific trigger for symptoms in a fifth subject. Diagnoses were confirmed by an ENT and neurologists in three patients. Two of the patients self-diagnosed with motion-triggered MdDS. Patients' self-scoring of their symptoms prior to treatment, on average, ranged from 3 to 6. All five patients reported improvement. On average, symptom improvement was 76% (range 60–100%). None of the patients reported any sign of motion sickness while using the device.

**Table 1:** Demographical data.

**Patient ID**	**Age**	**Trigger**	**MdDS duration**	**Score before**	**Score after**	**Score follow-up**
VRG017	50	stress	3 months	6	0	1.5
VRG018	39	cruise	1 month	4	0.5	3
VRG019	46	cruise	2 months	5	2	1
VRG020	26	boating	2 months	1–4	0–1	1–3
VRG021	48	long car ride	2 months	3–4	1	0–0.5

Among all phantom sensations of motion, bobbing is less common (2) and was not reported by any participants in this study (Table 2). Each patient reported a sensation of gravitational pull at least in one direction. The number of other motion symptoms did not correlate with MdDS severity. While patients VRG017 and VRG018 had all the symptoms besides bobbing, they scored their pretreatment symptom severity as 6 and 4, respectively. Patient VRG019 experienced only swaying but scored their pretreatment severity as 5. Two other patients had larger numbers of symptoms compared to those of VRG019 but had lower pretreatment scores. The same is true for post-treatment scores. While a significant improvement was reported, patient VRG019 had the highest post-treatment score compared to that of all other patients. Thus, the number of motion sensations experienced by individual patients did not correlate with symptom severity self-scores or with treatment effectiveness.

**Table 2:** Phantom sensations of motion experienced by patients.

**Patient ID**	**Rocking**	**Swaying**	**Bobbing**	**Trampoline walking**	**Gravitational pull**
VRG017	+	+	−	+	Yes, right, backward
VRG018	+	+	−	+	Yes, down, forward
VRG019	−	+	−	−	Yes, back
VRG020	+	−	−	+	Yes, right
VRG021	−	+	−	+	Yes, right

In addition to phantom motion sensations, patients experienced psychosomatic symptoms (Table 3). The number of psychosomatic symptoms reported by patients also did not correlate with symptom severity self-scores or with treatment effectiveness.

**Table 3:** Psychosomatic symptoms reported by patients.

**Patient ID**	**Brain fog**	**Fullness of ears**	**Heavy head**	**Heavy lags**	**Fuzzy vision**	**Fatigue**	**Sensitivity to fluorescent light**	**Sensitivity to computer screen**	**Unsteadiness in crowds**
VRG017	+	+	–	–	–	+	–	–	+
VRG018	–	+	+	+	+	–	–	+	–
VRG019	–	–	–	–	–	+	–	–	+
VRG020	–	+	+	–	+	–	+	–	–
VRG021	+	+	+	+	–	+	+	+	+

None of the patients in this study reported vection, which is frequently reported with full-field stimulation (2, 45).

### Effect of Treatment on MdDS Severity Self-Score

The average pretreatment symptom severity self-score was 4.2 ± 1.4 ranging from 1 to 6. In four patients, the severity level was consistent from day to day, and in one, it varied from 1 to 4 over the course of the day and from day to day (Table 1).

**Figure 1:**
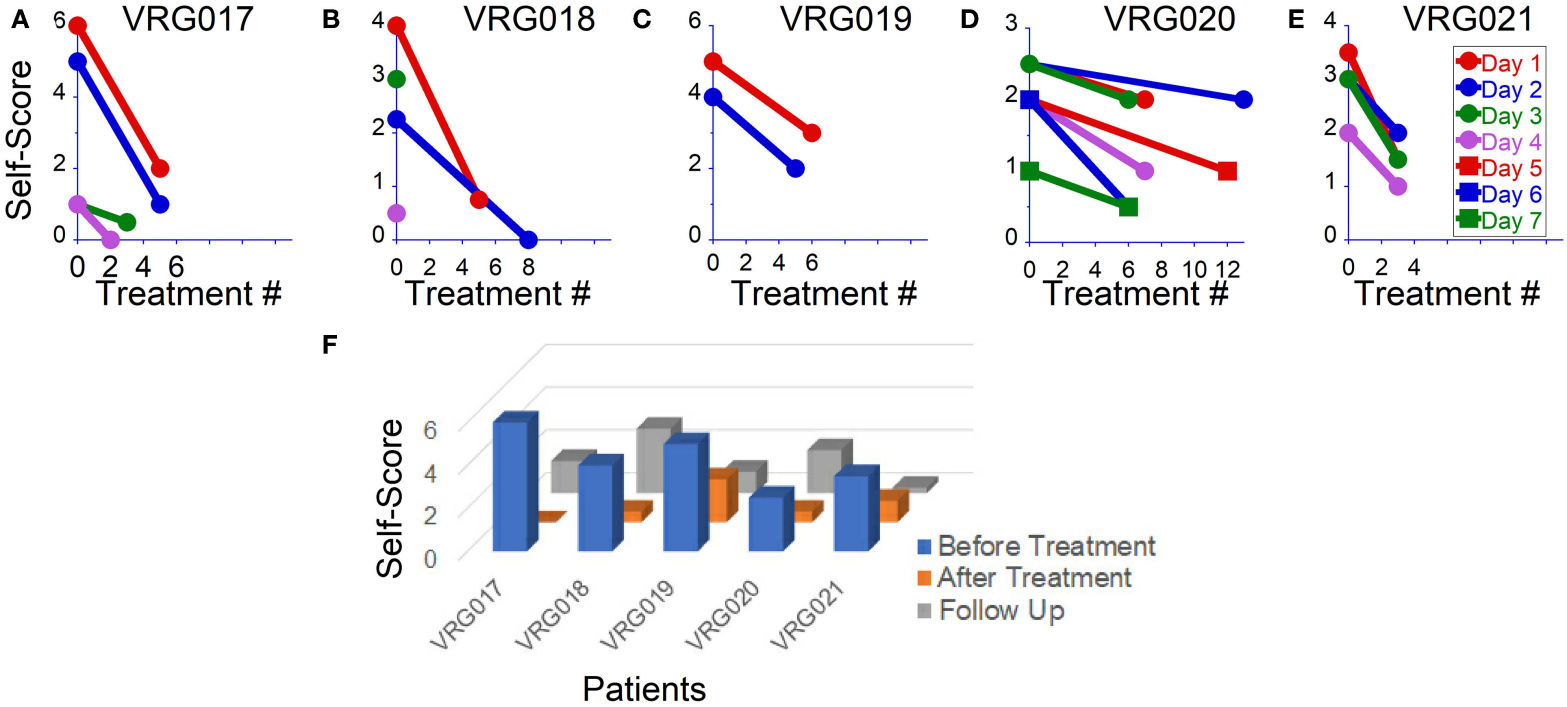
Overall symptom severity self-scores before and after readaptation treatment in five patients. **(A–E)** Scores after individual treatments in each patient. **(F)** Overall symptoms score obtained before, after and on follow-ups.

On the first day of examination, patient VRG017 reported sensations of rocking, swaying, and gravitational pull to the right. The patient could not maintain a standing upright posture with eyes closed; therefore, the Fukuda test could not be done. An OKN direction to the left for readaptation treatment was chosen based on gravitational pull to the right (2). The patient was first treated for gravitational pull and reported improvement after watching stripes going left for 2 min. After this treatment, post-urography revealed rocking at 0.1–0.2 Hz. The patient was treated for rocking at 0.2 Hz for 2 min and at 0.1 Hz for 3 min. After that, post-urography revealed gravitational pull back, which was treated by stripes going up for 2 min. Overall, on day 1, the patient reported significant symptom improvement from a score of 6–2 (Figure 1A, red circles). The next day, the patient reported that almost all symptoms returned back by 5 p.m. on day 1. Post-urography revealed small rocking at ≈0.4 Hz and swaying at ≈0.3 Hz, reduced sensation of pull to the right, and a strong pull back. The patient was treated for the backward pull for 13 min and reported significant improvement of all symptoms. Post-urography revealed small rocking at ≈0.2 Hz, and the patient was treated for that for 2 min. The patient reported some pull back, and the treatment for pull back was repeated for 5 min. Overall, the patient reported significant symptom decrease from a score of 5–1 on that day (Figure 1A, blue circles). On day 3, the patient reported that, regardless of all potential triggering activities that patient had on day 2, symptoms remained at score 1. Small gravitational pull back was treated for 4 and 5 min. After that, small rocking at ≈0.2 Hz was treated for 1 min. The patient reported further symptom improvement to score 0.5 on that day (**Figure 3A**, green circles). On day 4, the patient reported that symptoms stayed at a score of 0.5 since the last treatment. Post-urography revealed small rocking at ≈0.2 Hz or gravitational pull back. Treatment for pull back was performed for 5 min, and then that for rocking for 1 min. After that, the patient reported no symptoms (Figure 1A, pink circles). Thus, the possible reason for symptoms returning after day 1's treatments is because gravitational pull back, which was compensated by body tilting in the opposite direction, was interpreted and treated as rocking. After gravitational pull back was treated on days 2–4, symptom improvements immediately after treatment remained unaffected by daily activity (Figure 1A).

**Figure 2:**
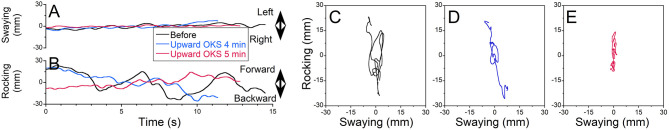
The static post-urography obtained from patient VGR018 standing with feet apart and eyes closed over 15 s. **(A)** Swaying. **(B)** Rocking (black trace, prior to treatment; blue, after treatment 1; red, after treatment 2). **(C–E)** *X*–*Y* plot of posture obtained prior to treatment **(C)** and after head motion side-to-side at 0.2 Hz for 2 min **(D)** and at 0.1 Hz for 3 min **(E)**.

Patient VRG018 reported symptoms of rocking, swaying, and trampoline walking, which intensified due to prolonged traveling. The patient was treated for these symptoms and reported improvement from a score of 4–0.5 on day 1 (Figure 1B, red symbols). Treatment, however, remained effective only for 2 h, after which symptoms returned to the baseline. In the morning on day 2, after a good night's sleep, symptoms were reduced to a score of 2. Treatment on day 2 brought the symptoms to score 0 (Figure 2B, blue traces). On day 3, the patient woke up with a migraine that brought symptoms to score 3 (Figure 1B, green symbol). It was recommended for the patient to stay home on that day. The next day, the patient called reporting very low symptoms at score 0.5 and refused to come in for further treatments (Figure 2B, purple circle).

**Figure 3:**
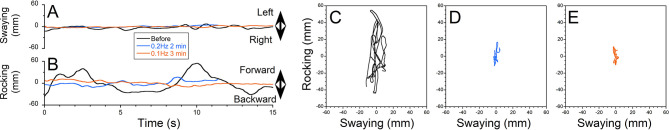
The treatment of gravitational pull-back sensations. Patient VRG017 had experienced gravitational pull back. **(A)** Swaying and **(B)** rocking during treatment. Resistance to pull back on the trace looks like body rocking at 0.2 Hz with constant body drift backward. **(C–E)** X-Y plot of posture obtained prior to treatment **(C)**, after two treatments with upward OKN for 4 min **(D)** and 5 min **(E)**, respectively.

Patient VRG019 experienced swaying at 0.25–0.31 Hz and bobbing sensations. The Fukuda test was inconclusive (right 45° once out of two tests). Post-urography revealed some gravitational pull back. The patient was treated for swaying by a head up–down motion at 0.3 Hz for 1 min with no effect and then for back pull for 1 min, with some improvement reported. After that, rocking rather than swaying was observed. The patient was treated for rocking at 0.2 and 0.25 Hz for 1 min each and reported only a small pull back that was treated for 2 min. Overall, the patient reported improvement from a score of 5–3 on day 1 (Figure 3C, red circles). The patient came back only 6 days later, when symptoms increased to a score of 4–5. Patient reported significant swaying, rocking, and pull back. The patient was treated for pull back for 3 min first and reported less sway and less gravitational pull. Then the patient was treated for rocking for 1 min with no effect. After that, OKN direction was reversed, and the patient was treated for rocking at 0.3, 0.1, and 0.05 Hz for 1, 3, and 5 min, respectively, and reported only a small pull back sensation. After pull back treatment for 5 min, overall symptoms decreased to a score of 2. The patient never came back for more treatments (Figure 1C, blue circles).

On the first day of examination, patient VRG021 had mild symptoms with a score of 1 while sitting but reported sensations of the ground moving side-to-side with a score of 2–4 when walking. Additionally, the patient reported pull forward and small pull right sensations. The Fukuda test was 20° to the left in both trials. Thus, pull right and Fukuda results contradicted in terms of best OKN direction for the treatment. There were no other clues to determine the direction of OKN. Because of this, the pull back was treated first for 1 min. After this, we treated swaying by an arbitrarily chosen OKN going left and head up–down motion at 0.2 Hz for 1 min. No changes were reported. Treatment was repeated with OKN going right. The patient reported some improvement. Treatments for swaying with OKN going right were repeated several times, and the patient reported significant improvement (Figure 1D, red symbols). Long driving (80 miles) back home induced some symptoms as well as an increased sensitivity to light. On day 2, treatment for pull forward was repeated three times for 2 min each. When swaying was treated with OKN to the right for 2 min, the patient's reported symptoms increased. Based on this report, OKN direction was changed to the left, and treatment for swaying was repeated nine times at frequencies 0.2 and 0.05 Hz. No improvement was reported (Figure 1D, blue circles). On day 3, treatment with OKN to the left was continued with no actual success (Figure 1D, green circles). On treatment day 4 (after the weekend), the patient reported virtually the same symptoms with the addition of a strong brain fog. Thus, OKN direction to the left was not effective. Treatment was repeated with OKN to the right, and significant improvements in brain fog and postural stability were reported (Figure 1D, pink circles). Treatment was continued with OKN to the right for 3 more days, with gradual symptom improvement on each sequential day (Figure 1D, red, blue, and green squares). Thus, OKN direction on days 1–3 was incorrect, and the treatment induced more symptoms. When OKN direction was reversed, the patient, similar to all other patients, reported gradual symptom improvement on each sequential day.

The last patient, VRG021, could not maintain a standing-upright posture with eyes closed; therefore, the Fukuda test was not done. The major symptoms on the first examination on day 1 were strong gravitational pull back and some pull to the right. The patient was treated repeatedly for pull back sensation for 2, 2, and 4 min, and improvement from a score of 3.5–1.5 was reported (Figure 1E, red circles). On day 2, the patient reported symptom improvement with some pull back and swaying. After 5 min of treatment for pull back, the patient reported small sway and pull right sensation. The patient was treated for both sensations consecutively and reported small symptom improvement from a score of 3–2 on that day (Figure 1E, blue circles). After that treatment, the patient felt improvement until a large crowd triggered some symptom back. Since the major symptom was the pull back sensation, on day 3, the patient was treated only for gravitational pull back for 10, 5, and 5 min, consecutively (Figure 1E, green circles). On day 4, the patient still had some gravitational pull back and a small sway. The patient was effectively treated for pull back for 10 and 5 min and in between was treated for sway with OKN going left for 1 min. The treatment for sway was not effective, but the treatment for pull back improved the patient's symptoms to a score of 1. When their average pretreatment score was compared to their last post-treatment score, all patients reported significant improvements (Figure 1F, blue vs. orange columns).

Treatment protocol in this study was similar to that used for readaptation treatment with full-field OKN (2). That is, we first targeted symptoms that bothered the patient the most. Typically, within one treatment day, symptoms dropped by at least 50%; however, the next day, patients may have had some symptoms rebound, although still below pretreatment levels. Improvement gradually accumulated over four treatment days.

Treatment for rocking was used 62 times for these five patients. In 35 cases (56%), patients reported symptom improvement. In 21 cases (34%), patients did not notice a difference, and in six cases (10%), patients reposted worsening of symptoms. All six worsening cases were reported by subject VRG029, when the sway sensation was treated.

The gravitational pull back sensation was treated 23 times. In 19 cases (83%), patients reported immediate improvement. In four cases (17%), patients were uncertain about the effect. The gravitational pull forward sensation was applied 10 times. In seven cases (70%), patients reported immediate improvement. In two cases (20%), there was uncertainty about treatment effect, and in one case (10%), a worsening of the symptoms was reported. The sideways pull was treated seven times. In five cases (71%), immediate improvement was reported, and in two cases (29%), patients did not note the difference.

### Follow-Up Study

Prior to treatment, only patient VRG017 did not report significant changes in symptoms with any daily activity. In the other four patients, symptoms could be elevated by prolonged traveling, fluorescent lights, flickering of computer screens, use of elevators and escalators, and exposure to crowds. After the treatment, patients VRG019 and VRG021 no longer reported strong symptom fluctuations by daily activity. The scores of all three patients remained low when followed up 4 ± 1 months after the treatment (Figure 1F, gray columns, Table 1).

In patient VRG018, prior to treatment, prolonged (>1 h) driving or taking the subway intensified symptoms. After the treatment, prolonged daily traveling remained a strong trigger. The patient was invited back to the lab 7 months after VR treatment and was treated for 2 days with full-field OKN. The treatment reduced symptoms from a score of 3–0.5, although the patient experienced worsening symptoms after traveling home. When followed up 11 months after VR treatment, the patient reported symptoms with a generally low score of 0 with occasional spikes to a score of 2. The patient also reported that since the last treatment, she is limiting daily driving to short distances only.

Patient VRG020 was also invited back 3 months after VR treatment and treated for 2 days with full-field OKN. Treatment was effective on each day, but sitting at work in front of a computer screen with strong fluorescent lights on reversed the treatment effects. On the last follow-up 9 months after VR treatment, the patient reported symptom improvement. At the same time, over the last month, the patient is self-quarantined at home and completely avoids fluorescent lights and long time spent in front of a computer screen.

Thus, the treatment remained effective in three out of five patients (60%) during normal daily activity and perhaps would be effective in all patients if specific triggers were avoided.

### Effect of Treatment on Static Posture

#### Treatment of Rocking

Figure 2 shows a typical example of rocking at ≈0.15 Hz experienced by MdDS patient VRG018 (Figures 2A,B, black traces). Rocking was first treated by activation of velocity storage with OKN to the left and moving the head side-to-side at 0.2 Hz for 2 min (Figures 2A,B, blue traces). Rocking was substantially reduced after the first treatment (Figure 1B). Patient was additionally treated for 3 min, while their head was moved side-to-side at 0.1 Hz (Figure 1B, red traces). Prior to treatment, static posturography revealed some swaying and substantial rocking (Figure 2C, xRMS = 3 mm, yRMS = 21, trace 20 s = 477 mm). After the first treatment at 0.2 Hz, both swaying and rocking were reduced (Figure 2D, xRMS = 1 mm, yRMS = 7, trace 20 s = 196 mm). The second treatment at 0.1 Hz only partially reduced body motion (Figure 2E, xRMS = 1 mm, yRMS = 4, trace 20 s = 180 mm). Thus, postural stability was improved by ≈65% (67, 67, and 60%) after the first treatment and 70% (67, 81, and 62%) after the second treatment. There was no further postural improvement after the second treatment (*p* = 0.18).

The postural stability was recorded on 21 occasions before and after treatment for rocking/swaying. The percentage changes in COP (xRMS, yRMS, and trace duration over 20 s) were computed and averaged. The average postural improvement was 6 ± 35% (ranging −98 to 53%).

#### Treatment of Gravitational Pull

Patient VRG017 reported persistent sensations of gravitational pull back. The static post-urography revealed cyclic body oscillations very similar to the rocking shown in Figures 2, 3. The average body position, however, drifted backward (Figures 3A,B, black trace). Additionally, the *X*–*Y* plot of COP revealed that as the body resisted the pull back sensation, it had some small sway (Figure 3C, xRMS = 2 mm, yRMS = 11 mm, trace 20 s = 259). Pull back sensation was first treated by the upward OKN for 4 min. The sensation of pulling backward was reduced but not eliminated (Figures 3A,B, blue traces). This is also seen in the COP plot: while the magnitude of sway reduced, the backward drift was slower but of the same approximate magnitude (Figure 3D, xRMS = 1 mm, yRMS = 6 mm, trace 20 s = 199 mm). Treatment was repeated for another 5 min. No pull back sensation was reported after the second treatment (Figures 3A,B, red traces). The COP plot confirms great reduction of gravitational pull back (Figure 3E, xRMS = 2 mm, yRMS = 5 mm, trace 20 s = 163 mm). Thus, average improvement of postural stability after the first and the second treatments was 39 and 30%, respectively (*p* = 0.999). Gravitational pull back is the most common sensation among all five MdDS patients (Table 2). Despite the fact that gravitational pull direction varies among the patients, it was effectively treated for all patients in this group. Sensation of swaying was reported by four patients and was successfully treated with VR goggles; however, static post-urography indicates that none of the patients sway without actual rocking.

The postural stability was recorded on 10 occasions after the treatment for pull back sensations and on two occasions for pull forward sensations. The average improvement was 32 ± 27% (ranging −3 to 89%) for pull back and 28% (ranging −13 to 68%) for pull forward treatments.

### Subjective and Objective Evidence of the Treatment's Effectiveness

After 33 individual treatments for gravitational pull, the postural data and change in subjective scores were available. The subjective changes in symptoms were compared with the percentage of average postural improvement. Average postural improvement was computed as an average improvement of three values: xRMS (%), yRMS (%), and trace 20 s (%). Results were categorized as follows: category 1, improvement ≥10%; category 0, abs[improvement] <10%; and category −1, worsening of posture > −10%. Similarly, subjective score was categorized as follows: category 1, symptom improvement; category 0, no changes; category −1, symptom worsening. No correlation was found between subjective and objective changes in symptoms. Data are summarized in a cross-tabulation (Table 4).

**Table 4:** Subjective vs. objective data cross-tabulation.

**Objective**					
Subjective		−1	0	1	Total
	−1	0	1	1	2
	0	4	5	3	12
	1	2	4	13	19
Total		6	10	17	33

Table 4 indicates that subjective and objective improvements were reported 19 and 17 times and coincide with 13 cases. Less coincidence was observed when no changes were reported and observed (5 out of 12 subjective and 5 out of 10 objective cases). Thus, in total, in 18 cases out of 33, objective and subjective symptom changes coincide. Because the sample size is too small, the chi-square test was insignificant (*p* = 0.167). Alternatively, this could be an indication of significant contribution of psychosomatic symptoms in overall scoring.

## Discussion

The purpose of this pilot project was to determine whether limited-field OKN produced by VR goggles may have clinical utility for treatment of MdDS. While the interpretation of the findings must be taken in the context of this not being a clinical trial, these data do suggest that further examination of a VOR readaptation protocol using VR goggles with a restricted visual field is warranted. This assertion is based on all five patients in this study showing positive responses to treatment with the limited-field OKN stimulation. Thus, limited-field OKN stimulation may be an effective stimulus for the activation of the velocity storage to the extent that it could be used for MdDS treatment.

Full-field OKN is believed to be the most efficient way to activate velocity storage (22, 55). Full-field stimulation is also the most efficient way to induce circular vection (46). Additionally, the amount of circular vection is correlated with the duration of OKAN (56), which is directly related to the activation of velocity storage (47, 57). Furthermore, OKAN can be induced by peripheral vision, while the central (foveal) vision is blocked or lessened (18, 56). Nevertheless, the central vision also plays a role, since OKN velocities are higher if central vision is activated (18), and velocity storage cannot be activated if OKN in the foveal area is in an alternate direction with the peripheral vision (56). Thus, OKS with a restricted visual field may not be as effective as a full-field OKN. While the size of the visual field is a factor (21), it is less relevant for low-speed OKN, which is commonly used for MdDS treatment (22, 23).

In the original readaptation paper, the authors argued that the sensation of self-motion (vection) could be critical for MdDS treatment (45). Vection is generated by activation of the peripheral vision (58). In the present study, visual periphery was not activated, and none of the five patients experienced vection, yet treatment remained effective. OKN and therefore velocity storage, however, appear to have been activated by a small visual field (21, 58). Thus, vection is not likely critical for MdDS treatment. Thus, this study indicates that lack of vection and limited size of the visual field are not crucial factors, in activation of velocity storage to the limit, necessary for MdDS treatment by VOR readaptation.

Over the last 5 years, more than 600 patients had been treated for MdDS at Mount Sinai. About half of them were from New York City or in driving distance from Mount Sinai. This indicates that several thousands of patients may suffer from MdDS across the United States and suggests that more treatment options need to be available. The current equipment used for MdDS treatment at Mount Sinai is bulky and expensive and cannot be purchased for immediate use since it requires several substantial modifications. Thus, this treatment could not be easily replicated in its present form. VR goggles, however, are commercially available, are relatively inexpensive, and could provide a uniform environment for use in multiple laboratories to study the treatment effectiveness of VOR readaptation protocols for MdDS. The effectiveness of VR goggles vs. full-field OKN needs to be further investigated, though this study demonstrates the feasibility of this approach.

One of the difficulties of the proposed approach is that each patient has a unique set of symptoms, which require a unique treatment protocol. Figure 3 demonstrate the complexity of the approach. Not only could the symptoms vary on different days or after individual treatments, but success is also dependent on whether the correct treatment is performed at the right time. The same treatment could be very effective at the beginning, but not later in the day. For example, rocking was eliminated on day 1, but gravitational pull sensation remained strong. When gravitational pull was eliminated on day 2, it had a long-lasting effect on the patient's symptoms. Some of the patients treated at Mount Sinai used an OKN web-based application to reduce their symptoms at home in case they were retriggered (http://mdds.nyc/okn-stripes-visualization/). The treatment is based on the protocol that was developed during in-lab testing. The home setup requires the computer to be connected to a large-screen TV or short-throw video projector. The effectiveness of the home treatment, however, was largely varied based on each individual home setup. The VR goggles used in this study is a promising approach for MdDS self-treatment at home.

It has been reported that scrolling on a computer screen or just watching a cellphone screen is frequently a trigger for MdDS symptoms (2). While visual sensitivity to screens was not measured in this study, it should be noted that none of the patients reported any discomfort while watching the OKN stripes in this study. However, this could be because our small sample size did not include any patients with high visual sensitivity. Thus, further testing of VR goggles is required on larger populations of MdDS patients to determine how flickering of the cell phone screen affects MdDS patients with heightened visual sensitivity.

Thus, the limitation of this study is the small and not randomly selected group of patients. Our patient group may not include all types of MdDS patients who were treated with full-field stimulation in terms of sensitivity to visual motion, sensitivity to brightness of the screen, motion sickness, anxiety, claustrophobia, etc. Due to the individual variabilities and drop-off from the study, the protocols of each patient are different. Thus, this study is lacking comparison between full-field and limited-field stimulation groups; there was no sham/placebo; there was no blinding/masking. To eliminate potential problems, a larger study of MdDS patients' treatment using VR goggles vs. full-field stimulation is required.

In conclusion, MdDS is composed of constant phantom sensations of motion, which are frequently accompanied by increased sensitivity to light, inability to walk on a patterned floor, the sensation of ear fullness, head pressure, anxiety, and depression. It has been previously hypothesized that MdDS is the result of maladaptive changes in the polysynaptic VOR called velocity storage. Past research indicates that full-field OKS is an optimal way to activate velocity storage. Unfortunately, the full-field OKS devices are typically bulky and not commonly available. We questioned whether VR goggles with a restricted visual field could effectively simulate a laboratory OKS environment for MdDS treatment. While the interpretation of our findings must be taken in the context of the limitations of the study, these data do suggest that further examination of a VOR readaptation protocol using VR goggles with a restricted visual field is warranted.

## Data Availability Statement

The raw data supporting the conclusions of this article will be made available by the authors, without undue reservation.

## Ethics Statement

The studies involving human participants were reviewed and approved by Mount Sinai IRB. The patients/participants provided their written informed consent to participate in this study.

## Author Contributions

SY provided patient treatment, data analyses, and wrote the initial draft of the manuscript. RZ, BC, and CL designed and developed Virtual Reality App. All authors equally contributed to writing the final version of this manuscript.

## Conflict of Interest

The authors declare that the research was conducted in the absence of any commercial or financial relationships that could be construed as a potential conflict of interest.
